# Outcomes of Simulation-Based Education for Vascular Access: A Systematic Review and Meta-Analysis

**DOI:** 10.7759/cureus.17188

**Published:** 2021-08-15

**Authors:** Hiromu Okano, Takuya Mayumi, Yuki Kataoka, Masahiro Banno, Yasushi Tsujimoto, Akihiro Shiroshita, Shunsuke Taito, Joho Tokumine

**Affiliations:** 1 Department of Critical Care and Emergency Medicine, National Hospital Organization Yokohama Medical Center, Yokohama, JPN; 2 Department of Cardiovascular Medicine, Kanazawa University, Kanazawa, JPN; 3 Department of Internal Medicine, Kyoto Min-Iren Asukai Hospital, Kyoto, JPN; 4 Department of Systematic Reviewers, Systematic Review Workshop Peer Support Group (SRWS-PSG), Osaka, JPN; 5 Department of Psychiatry, Nagoya University Graduate School of Medicine, Nagoya, JPN; 6 Department of Psychiatry, Seichiryo Hospital, Nagoya, JPN; 7 Department of Healthcare Epidemiology, Graduate School of Medicine and Public Health, Kyoto University, Kyoto, JPN; 8 Department of Respiratory Medicine, Ichinomiyanishi Hospital, Ichinomiya, JPN; 9 Division of Rehabilitation, Hiroshima University Hospital, Hiroshima, JPN; 10 Department of Anesthesiology, Kyorin University School of Medicine, Tokyo, JPN

**Keywords:** central venous catheter, picc, radial arterial catheter, dialysis catheter, simulation training

## Abstract

Simulation training is key to developing skills for vascular access. However, the efficacy of simulation-based education remains unclear. We conducted a well-designed and updated systematic review to investigate the efficacy of these programs. Randomized controlled trials (RCTs) were researched using the following databases from inception until July 26, 2020: MEDLINE, Embase, the Cochrane Central Register of Controlled Trials (CENTRAL), Education Resources Information Center (ERIC), Cumulative Index to Nursing and Allied Health Literature (CINAHL), ClinicalTrials.gov, and International Clinical Trials Registry Platform (ICTRP). RCTs included patients undergoing insertion of central venous catheters (CVCs), peripherally inserted central catheters (PICCs), and radial arterial catheters. We compared the group that received simulation training with the group that received traditional training. We also assessed the success rate, adverse events, and first-attempt success using a random-effects meta-analysis. The protocol was registered at Protocols.io (dx.doi.org/10.17504/protocols.io.biu6keze).

Seven RCTs (n=866) were evaluated. The meta-analysis showed that simulation-based education increased the overall success rate compared with traditional education (risk ratio: 1.08, 95% CI: 1.03 to 1.13; six RCTs; 840 participants; I^2^=0%; moderate certainty of evidence). However, it was unclear whether or not simulation-based education had an effect on reducing adverse events when compared with traditional education (risk ratio: 1.00, 95% CI: 0.63 to 1.58; five studies; 750 participants; I^2^=37%; very low certainty of evidence) or on raising first-attempt success rates (risk ratio: 1.34, 95% CI: 0.93 to 1.94; three studies; 244 participants; I^2^=59%; very low certainty of evidence).

Simulation-based education may help develop skills for successful vascular access. However, it is unclear whether simulation-based education actually reduces the incidence of adverse events. Fine control of the needle tip is probably necessary to prevent adverse events. Simulation-based education might be required in the future for outcome-based task training.

## Introduction and background

Vascular access under ultrasound guidance has gained wide popularity in the practice of medicine. This procedure enables catheterization under real-time observation. However, the efficacy of ultrasound-guided vascular access is limited as it requires proficiency in ultrasound. Central venous catheterization may cause lethal adverse events, including mechanical complications (pneumothorax, hemothorax, and airway occlusion due to a large neck hematoma causing bleeding from the injured artery) and catheter-related bloodstream infections. Ultrasound guidance for central venous catheterization is expected to prevent these lethal mechanical complications [1]. A peripherally inserted central catheter (PICC) is a safer central venous catheter (CVC) than a conventional CVC due to the reduced risk of lethal complications. However, the target vein for PICC is smaller than that for conventional CVCs. Therefore, the required skillset for inserting a PICC may be more complex than that for conventional central venous catheterization. Radial artery catheterization is also as difficult as PICC due to the small target vessel, the radial artery.

Successful vascular access may require appropriate hands-on simulation training before starting on-the-job training in a clinical setting [2]. However, it is difficult to assess what an appropriate simulation-based education entails. If there is an appropriate simulation-based education, it may result in a good outcome in a clinical setting. The purpose of this systematic review was to assess whether simulation-based education for vascular access improved the success rate and decreased complication rates compared to traditional education.

## Review

Methods 

Protocol and Registration

This systematic review was conducted in accordance with the Preferred Reporting Items for Systematic Reviews and Meta-Analyses (PRISMA) 2009 guidelines [3]. The protocol is registered (dx.doi.org/10.17504/protocols.io.biu6keze).

Research Question

Does simulation-based education for vascular access improve the success rate and decrease the complication rate compared with traditional education using on-the-job training?

Inclusion Criteria

The following criteria were used in the meta-analysis:

Type of study: randomized controlled trials (RCTs) were included irrespective of the publication status (including published and unpublished articles, conference abstracts, and letters), language, and country where the study was conducted. Population: (1) all patients who underwent procedures for vascular access, and (2) the types of vascular catheters used were CVCs, PICCs, and arterial catheters. Intervention: simulation training compared to traditional training. The primary outcomes were as follows: (1) the success rate, defined as the number of successful punctures divided by the number of punctured patients, and (2) adverse events as defined by the authors of the individual studies. The secondary outcome was the first-attempt success rate in eligible patients, defined as the number of successful punctures in the first attempt divided by the number of punctured patients.

Exclusion Criteria

Studies conducted by crossover, cluster randomization, or quasi-experimental methods were excluded. The observational term for evaluating the outcome was not considered an exclusion criterion. No exclusions were made based on the experience or occupation of the person performing vascular access. The type of catheter used in the simulation training was the same as that used in actual patients. The content of the simulation training was not considered an exclusion criterion. 

Search Strategy 

Databases used for the search were the Cochrane Central Register of Controlled Trials (CENTRAL; Supplemental Appendix 1), MEDLINE (via PubMed, Supplemental Appendix 2), EMBASE (Supplemental Appendix 3), Education Resources Information Center (ERIC; Supplemental Appendix 4), and Cumulative Index to Nursing and Allied Health Literature (CINAHL; Supplemental Appendix 5). Further searches for ongoing and unpublished studies were performed using ClinicalTrials.gov and the International Clinical Trials Registry Platform (ICTRP). A literature review was conducted using these electronic databases from inception until July 26, 2020.

Selection of the Studies and Data Extraction

Two review authors (TM, HO) independently reviewed the titles and abstracts during the first screening. The full texts were reviewed at the second screening, and data extracted from the studies were transferred into standardized data recording forms. If there was a discrepancy between the two review authors, an agreement was reached through discussion. If the conflict could not be resolved after a discussion between the two reviewers, a third reviewer would be consulted to resolve the conflict. In addition, if data were lacking, we contacted the authors of the original study. If the studies had only an abstract and the review authors could not evaluate whether they met the review criteria, the review authors would contact the original study authors. The analysis was performed with available data if the authors of the original study could not be contacted. 

Quality Assessment

The risk of bias in the studies was assessed independently by two review authors (TM, HO) using the Cochrane Risk of Bias 2.0 tool for the following six domains: (a) bias arising from the randomization process, (b) bias due to deviations from intended interventions, (c) bias due to missing outcome data, (d) bias in the measurement of the outcome, (e) bias in the selection of the reported result, and (f) overall bias. If there was a discrepancy between the two review authors, an agreement was reached through discussion. If the conflict could not be resolved after a discussion between the two reviewers, the third reviewer would be consulted. Each domain was classified into one of the three following categories: high risk, low risk, and some concerns. The clinical trial sites (ClinicalTrials.gov, ICTRP) were used to evaluate publication bias. Publication bias was also assessed using funnel plots and Egger’s test.

Statistical Analysis

Statistical analyses were performed using the statistical software RevMan 5.4 (Cochrane, London, UK). A meta-analysis of risk ratios with 95% CIs was conducted for binary variables. The analysis was performed using a random-effects model. Heterogeneity was tested using a weighted Mantel-Haenszel χ^2^ test and quantified using the I^2^ statistic. I^2^ values of 25%-50% indicated low heterogeneity, 50%-75% indicated moderate heterogeneity, and >75% indicated high heterogeneity. A value >50% may be considered substantial heterogeneity [4].

Subgroup Analysis

Subgroup analyses were conducted to assess the heterogeneity of the clinical study participants and interventions. The first category of subgroups was catheter type, which included CVCs, peripherally inserted catheters, dialysis catheters, and arterial catheters. The second category included different CVCs and dialysis catheters, such as the internal jugular venous catheters, the subclavian venous catheters, and the femoral venous catheters. The third category involved different operators, such as doctors, nurses, and other healthcare providers.

Grading the Quality of the Evidence

Quality assessment was performed using the Grading Recommendations, Assessment, Development, and Evaluation (GRADE) approach for the following domains: risk of bias, inconsistency, imprecision, and publication bias. These were classified as very low, low, moderate, or high [5]. Our findings are indicated in Table 2. We included an overall grading of the certainty of the evidence for each of the primary outcomes. We also included the absolute effect/1,000 using the median event rate of control groups in the included studies, evaluated using the GRADE approach.

Difference Between Protocol and Review

Differences between the protocol and the research performed in this study were noted. The protocol stated that if a conflict could not be resolved after a discussion between the two reviewers, a third reviewer would be consulted to resolve the conflict. However, the conflicts were resolved by discussion between the two reviewers, and the third reviewer was not consulted. In addition, the subgroup analyses of catheter types were conducted without the dialysis catheter due to the lack of randomization of the dialysis catheter. Also, the study participants were all physicians, and no additional subgroup analysis was performed.

Results

**Figure 1:**
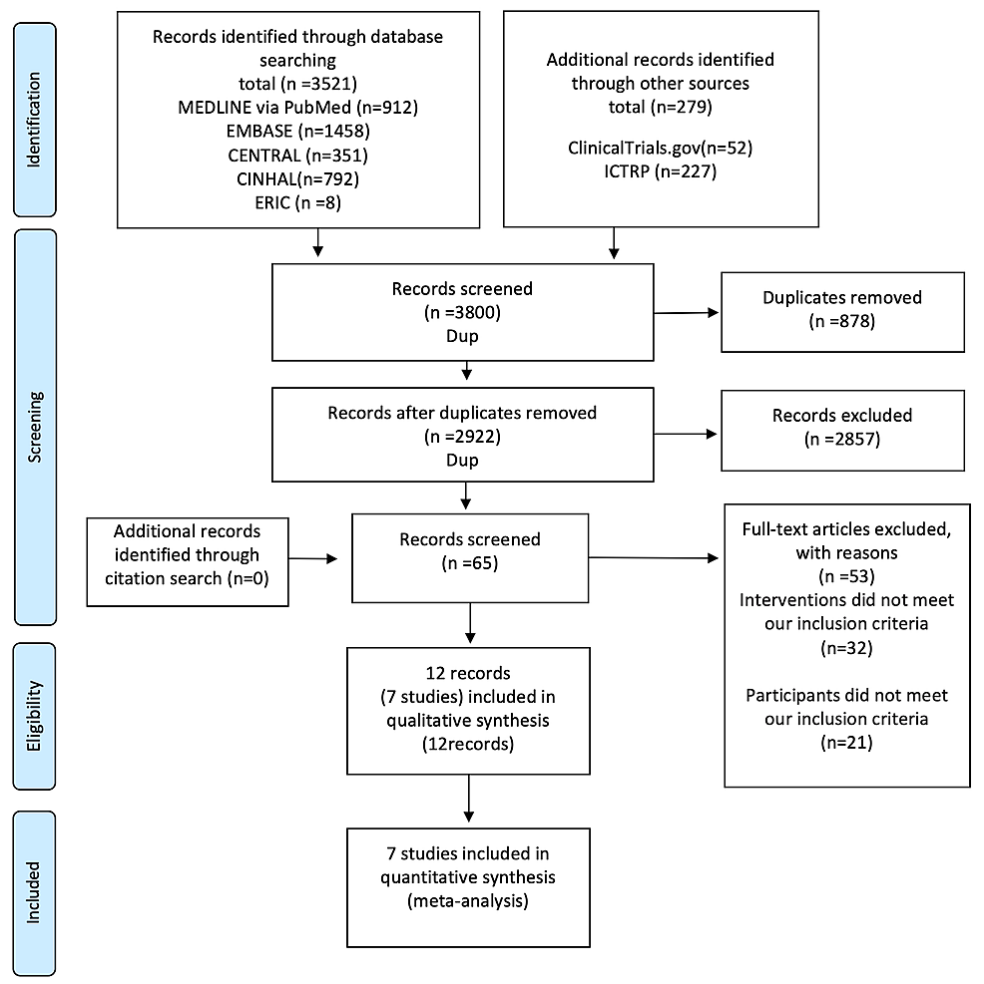
PRISMA 2009 flow diagram PRISMA: Preferred Reporting Items for Systematic Reviews and Meta-Analyses; CENTRAL: Cochrane Central Register of Controlled Trials; CINAHL: Cumulative Index to Nursing and Allied Health Literature; ERIC: Education Resources Information Center; ICTRP: International Clinical Trials Registry Platform

The process of the selection of the studies is shown in the PRISMA flow diagram (Figure 1).

Initially, 3,521 articles from the databases and 279 articles from other sources were identified. After removing the duplicates, 2,922 articles remained after the first screening. Subsequently, 65 articles with full text were retained by the second screening. Furthermore, 53 articles were removed based on the exclusion criteria. Finally, seven RCTs [6-12] (866 participants) consisting of 12 articles were included in the qualitative and quantitative synthesis (meta-analysis). The characteristics of the individual studies included in this meta-analysis are presented in Table 1.

**Table 1:** Characteristics of the studies CVC: central venous catheter; PICC: peripherally inserted central catheter; IJV: internal jugular vein; SV: subclavian vein; FV: femoral vein; PGY: postgraduate year

Study	Sample size (Sim/App)	Catheter	Placement	Participant	Instructor	Teaching method	Ultrasound
Velmahos et al., 2004 [6]	12/14	CVC	IJV	PGY1	Experts	Sim/App	No
Britt et al., 2009 [7]	34/39	CVC	IJV, SV	Junior residents	PGY4 residents, trauma fellows, or surgical critical care attending	Lec, Sim/Lec, App	No
Evans et al., 2010 [8]	246/249	CVC	IJV, SV, FV	PGY1, PGY2	Attending physicians, fellows, or senior residents	Sim/App	Yes
Smith et al., 2010 [9]	34/35	CVC	IJV	PGY1, PGY2	Trained faculty	Lec, Sim/Lec, App	No
Andreatta et al., 2011 [10]	16/16	PICC	Not mentioned	PGY1, PGY2	Anesthesiology attending	Lec, Sim/Lec, App	Yes
Peltan et al., 2015 [11]	49/38	CVC	IJV	Medicine interns	Pulmonary and critical care or emergency medicine attending physician	Sim/App	Yes
Oh et al., 2020 [12]	44/40	Arterial catheter	Radial artery	Anesthesiology residents (1–3 training years)	Unclear	Sim/Lec, App	Yes

**Figure 2:**
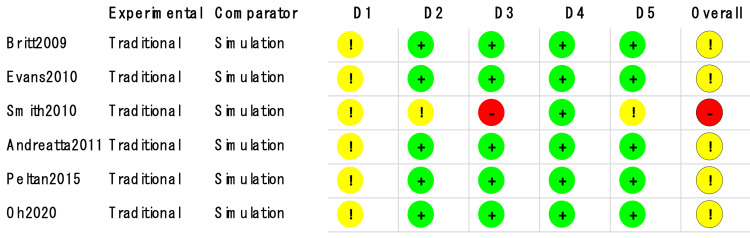
Methodological quality of trials using the Cochrane risk of bias 2 tool for overall success Symbols show a low risk of bias (+), some concerns (!), or a high risk of bias (-) D1: randomization process; D2: deviations from the intended interventions; D3: missing outcome data; D4: measurement of the outcome; D5: selection of the reported result

**Figure 3:**
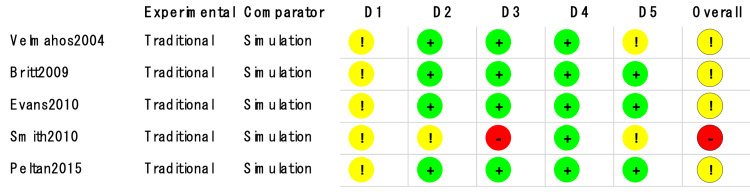
Methodological quality of trials using the Cochrane risk of bias 2 tool for adverse events Symbols show a low risk of bias (+), some concerns (!), or a high risk of bias (-) D1: randomization process; D2: deviations from the intended interventions; D3: missing outcome data; D4: measurement of the outcome; D5: selection of the reported result

**Figure 4:**
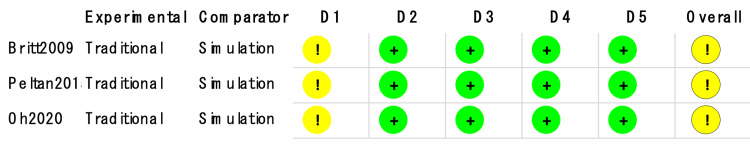
Methodological quality of trials using the Cochrane risk of bias 2 tool for first-attempt success Symbols show a low risk of bias (+), some concerns (!), or a high risk of bias (-) D1: randomization process; D2: deviations from the intended interventions; D3: missing outcome data; D4: measurement of the outcome; D5: selection of the reported result

Ultrasound was used in four of the studies [8,10-12]. Partial-task trainers were used in all studies. All studies also stated that complications did occur. Five studies [6-9,11] (n=750) compared CVC simulation-based education with traditional education. Of the remaining studies, one [10] (n=32) examined PICC, while the other (n=84) examined radial arterial catheterization [12]. Most of the studies had some concerns about the overall risk of bias, and one RCT [9] had a high overall risk of bias. The risk of summary bias in individual studies is shown in Figures 2, 3, 4.

Primary Outcome

**Figure 5:**
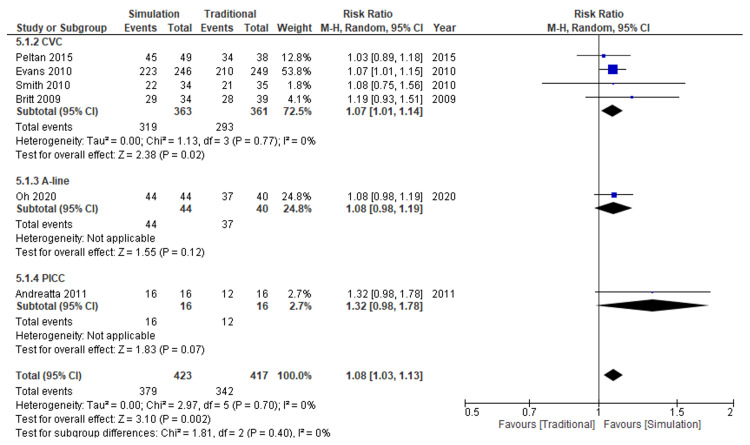
Forest plot comparison of overall success for all types of catheters Simulation versus traditional methods CI: confidence interval; M-H: Mantel-Haenszel

Six RCTs [7-12] that recruited 840 participants showed that simulation-based education might increase the overall success rate compared with traditional education (risk ratio: 1.08, 95% CI: 1.03 to 1.13; I^2^=0%; moderate certainty of evidence) (Figure 5).

Compared with traditional education, the absolute effect of simulation-based education on success rates was 66 more successes per 1,000 attempts (from 25 to 107 more) (Table 2).

**Table 2:** The GRADE quality assessment findings from seven RCT trials Overview of study design: patients or study population: (1) all patients underwent vascular access, and (2) the types of vascular catheters used were either central venous catheters, peripherally inserted central venous catheters, and arterial catheters. Setting: Any. Intervention: simulation training. Comparison with: traditional training ^a^Downgraded one level for serious limitations on study design (methods of sequence generation, allocation concealment, and masking poorly reported); ^b^downgraded one level as the direction of the result is not consistent; ^c^downgraded one level as the results span a clinically important threshold CI: confidence interval; GRADE: Grading Recommendations, Assessment, Development, and Evaluation; RCT: randomized controlled trial; RR: risk ratio

Quality assessment findings					
Outcomes	Anticipated absolute effects (95% CI)		Relative effect (95% CI)	No. of participants (studies)	Certainty of the evidence (GRADE)
	Risks with traditional education	Risks with simulation-based education			
Success	820 per 1,000	886 per 1,000 (845 to 927)	RR: 1.08 (1.03 to 1.13)	840 (6 RCTs)	⨁⨁⨁◯ MODERATE^a^
Complications	173 per 1,000	173 per 1,000 (109 to 274)	RR: 1.00 (0.63 to 1.58)	750 (5 RCTs)	⨁◯◯◯ VERY LOW^a,b,c^
First-attempt success	470 per 1,000	630 per 1,000 (437 to 912)	RR: 1.34 (0.93 to 1.94)	244 (3 RCTs)	⨁◯◯◯ VERY LOW^a,b,c^

**Figure 6:**
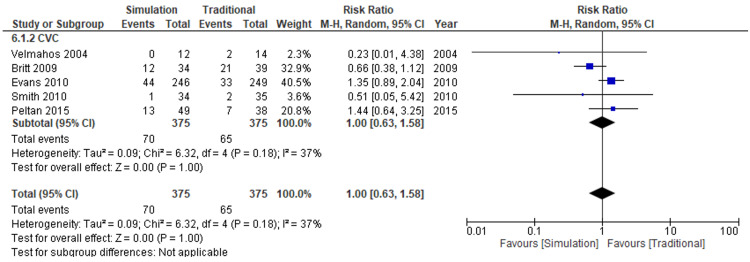
Forest plot comparison of adverse events for all types of catheters Simulation versus traditional methods CI: confidence interval; M-H: Mantel-Haenszel

The outcome of the complication rate between simulation-based education and traditional education was very uncertain (five studies, 750 participants, risk ratio: 1.00, 95% CI: 0.63 to 1.58; I^2^=37%; very low certainty of evidence) (Figure 6). Compared with traditional education, the absolute effect for complication rates of simulation-based education was five more per 1,000 attempts (from 56 to 97 more) (Table 2). 

Subgroup Analysis

The subgroup analysis for success rates showed no plausible heterogeneity regarding the types of catheters (test for subgroup difference, p=0.40) (Figures 5, 6).

Studies on radial artery catheters [12] and PICC [10] did not describe the outcome of adverse events. Hence, a subgroup analysis of adverse events could not be conducted.

Secondary Outcome

**Figure 7:**
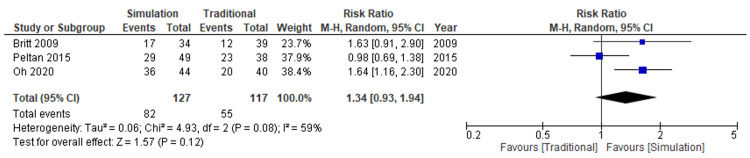
Forest plot comparison of first-attempt success for all catheters Simulation versus traditional methods CI: confidence interval; M-H: Mantel-Haenszel

The evidence of first-attempt success rate was unclear regarding the effect of simulation-based education compared with traditional education (three studies, 244 participants, risk ratio: 1.34, 95% CI: 0.93 to 1.94; I^2^=59%; very low certainty of evidence) (Figure 7).

Compared to traditional education, the absolute effect of simulation-based education for first-attempt success was 160 more successes per 1,000 attempts (from 33 to 442 more) (Table 2).

Discussion

There are two previous reviews [13,14] concerning the clinical outcomes of simulation-based education for CVC. A meta-analysis conducted by Ma et al. [13] evaluated 20 studies, of which four trials had assessed clinical outcomes after simulation-based education. They showed that simulation-based education improved learners’ performance and reduced the incidence of pneumothorax. Their review included two cohort studies [15,16]. In the subgroup analysis without cohort studies, simulation-based education did not affect the number of punctures or the incidence of adverse events. Another study by Madenci et al. [14] also assessed the same two cohort studies as in Ma et al.'s study. In our study, seven RCTs [6-12] were selected, five [6-10] of which were the same as those included in the study by Madenci et al. in 2014 [14]. We also included two new studies [11,12], of which one explored CVC [11] and the other [12] focused on radial artery catheterization. Our study aimed to evaluate the effect of simulation-based education on all vascular accesses by selecting only high-quality RCTs. This review showed that simulation-based education might increase the overall success rate compared to traditional education. However, we could not confirm the utilization of simulation-based education in reducing adverse events and improving first-attempt success rates.

We used rigorous methodology and followed standard guidelines to perform the systematic review [3-5,17]. However, we also acknowledge several limitations. Firstly, there was insufficient information about the study design (methods of sequence generation, allocation concealment, and reporting of blinding) and subgroup data in these studies. Although we contacted the authors to obtain as much of the missing information as possible, we could neither explicitly judge the risk of bias nor perform several pre-specified analyses. Second, only 40% of CVC studies reported the use of ultrasound in educational intervention, which might lower the generalizability of our findings. Third, in two studies [7,8], even though we asked the authors about the insertion site of the CVC as it may contribute to adverse events, we did not receive a reply. Hence, we could not conduct a subgroup analysis because of the missing data. Finally, in the current study, we were unable to determine the quality of the simulation-based education as the primary outcomes of each study varied. It is unclear whether the training provided in each study was sufficient to achieve the goals set by the respective authors in each study.

Ultrasound imaging visualizes the target vein, surrounding arteries, and organs. Hence, practice does not seem to be necessary. However, simulation training is necessary for safe ultrasound-guided vascular access [2]. The results of our study confirmed this assumption. Logically, increasing success rates should be associated with decreasing adverse event rates. This idea is based on the fact that multiple punctures tend to increase the incidence rate of mechanical complications [18]. However, the alternative hypothesis that reducing the number of punctures reduces the mechanical complication rate has not been proven. Therefore, we aimed to understand why mechanical complications occur during internal jugular venous catheterization. Mechanical complications may occur if the needle progresses unexpectedly or completely penetrates the internal jugular vein [19]. On the other hand, in the placement of the PICC and radial artery catheter, penetrating their target vessels may cause a hematoma, but lethal complications are rare. However, hematoma may inhibit successful catheterization in the placement of a PICC or radial arterial catheter. Hence, precise and fine control of the needle tip may increase the success rate and reduce mechanical complications in all catheterization procedures.

## Conclusions

Simulation-based education for vascular access may improve overall success rates. However, it is unclear whether it can reduce adverse events, including mechanical complications. If current simulation-based education contributes to patient safety, further improvements may be needed in the form of outcome-based task training programs. Further research on the learning curve of vascular catheterization will be key to the improvement of simulation-based education.

## Appendices

Supplemental file

Appendix 1: CENTRAL Search Strategy

(((((((([mh catheterization] OR [mh catheters]) OR [mh "catheterization, central venous"]) OR [mh "catheterization, peripheral"]) OR CVC:ti,ab) OR "peripherally inserted central catheter":ti,ab) OR PICC:ti,ab) OR "arterial line":ti,ab) AND ((((((([mh "educational status"] OR ([mh "educational status"] OR [mh Education])) OR [mh "simulation training"]) OR [mh "educational measurement"]) OR simulated:ti,ab) OR simulation:ti,ab) OR Education:ti,ab) OR [mh "task performance and analysis"])).

Appendix 2: MEDLINE (via PubMed) Search Strategy

(((((((("catheterization"[MeSH Terms] OR "catheters"[MeSH Terms]) OR "catheterization, central venous"[MeSH Terms]) OR "catheterization, peripheral"[MeSH Terms]) OR "CVC"[Title/Abstract]) OR "peripherally inserted central catheter"[Title/Abstract]) OR "PICC"[Title/Abstract]) OR "arterial line"[Title/Abstract]) AND ((((((("educational status"[MeSH Terms] OR ("educational status"[MeSH Terms] OR "Education"[MeSH Terms])) OR "simulation training"[MeSH Terms]) OR "educational measurement"[MeSH Terms]) OR "simulated"[Title/Abstract]) OR "simulation"[Title/Abstract]) OR "Education"[Title/Abstract]) OR "task performance and analysis"[MeSH Terms])) AND (((((((("randomized controlled trial"[Publication Type] OR "controlled clinical trial"[Publication Type]) OR "randomized"[Title/Abstract]) OR "drug therapy"[MeSH Subheading]) OR "placebo"[Title/Abstract]) OR "randomly"[Title/Abstract]) OR "trial"[Title/Abstract]) OR "groups"[Title/Abstract]) NOT ("animals"[MeSH Terms] NOT "humans"[MeSH Terms])).

Appendix 3: EMBASE Search Strategy 

S1        "EMB.EXACT.EXPLODE("catheterization")

S2        "EMB.EXACT.EXPLODE("catheter")

S3        EMB.EXACT("central venous catheterization")

S4        "EMB.EXACT.EXPLODE("central venous catheter")

S5        ab(CVC) OR ti(CVC)

S6        ab(peripherally inserted central catheter) OR ti(peripherally inserted central catheter)

S7        ab(PICC) OR ti(PICC)

S8        ab(arterial line) OR ti(arterial line)

S9        S1 OR S2 OR S4 OR S5 OR S6 OR S7 OR S8

S10      "EMB.EXACT.EXPLODE("educational status")

S11      "EMB.EXACT.EXPLODE("education")

S12      "EMB.EXACT.EXPLODE("simulation training")

S13      "EMB.EXACT.EXPLODE("task performance")

S14      ab(simulated) OR ti(simulated)

S15      ab(simulation) OR ti(simulation)

S16      ab(Education) OR ti(Education)

S17      S10 OR S11 OR S12 OR S13 OR S14 OR S15 OR S16

S18      (ab(random*) OR ti(random*)) OR (ab(clinical NEAR/1 trial*) OR ti(clinical NEAR/1 trial*)) OR (EMB.EXACT("health care quality"))

S19      S9 AND S17 AND S18

Appendix 4: ERIC

(((((((((MH "catheterization+") OR (MH "catheters+")) OR (MH "catheterization, central venous+")) OR (MH "catheterization, peripheral+")) OR TI CVC OR AB CVC) OR TI "peripherally inserted central catheter" OR AB "peripherally inserted central catheter") OR TI PICC OR AB PICC) OR TI "arterial line" OR AB "arterial line") AND ((((((((MH "educational status+") OR ((MH "educational status+") OR (MH "Education+"))) OR (MH "simulation training+")) OR (MH "educational measurement+")) OR TI simulated OR AB simulated) OR TI simulation OR AB simulation) OR TI Education OR AB Education) OR (MH "task performance and analysis+"))).

Appendix 5: CINAHL

(((((((((MH "catheterization+") OR (MH "catheters+")) OR (MH "catheterization, central venous+")) OR (MH "catheterization, peripheral+")) OR TI CVC OR AB CVC) OR TI "peripherally inserted central catheter" OR AB "peripherally inserted central catheter") OR TI PICC OR AB PICC) OR TI "arterial line" OR AB "arterial line") AND ((((((((MH "educational status+") OR ((MH "educational status+") OR (MH "Education+"))) OR (MH "simulation training+")) OR (MH "educational measurement+")) OR TI simulated OR AB simulated) OR TI simulation OR AB simulation) OR TI Education OR AB Education) OR (MH "task performance and analysis+"))) AND (((MH randomized controlled trials) OR (MH double-blind studies) OR (MH single-blind studies) OR (MH random assignment) OR (MH pretest-posttest design) OR (MH cluster sample) OR (TI (randomised OR randomized)) OR (AB (random*)) OR (TI (trial)) OR (MH (sample size) AND AB (assigned OR allocated OR control)) OR (MH (placebos)) OR (PT (randomized controlled trial)) OR (AB (control W5 group)) OR (MH (crossover design) OR MH (comparative studies))))NOT ((((MH animals+) OR (MH (animal studies)) OR (TI (animal model*))) NOT (MH (human)))).

## References

[1] Rupp SM, Apfelbaum JL, Blitt C (2012). Practice guidelines for central venous access: a report by the American Society of Anesthesiologists Task Force on Central Venous Access. Anesthesiology.

[2] Moureau N, Lamperti M, Kelly LJ, Dawson R, Elbarbary M, van Boxtel AJ, Pittiruti M (2013). Evidence-based consensus on the insertion of central venous access devices: definition of minimal requirements for training. Br J Anaesth.

[3] Moher D, Liberati A, Tetzlaff J, Altman DG (2009). Preferred reporting items for systematic reviews and meta-analyses: the PRISMA statement. PLoS Med.

[4] Sterne JA, Savović J, Page MJ (2019). RoB 2: a revised tool for assessing risk of bias in randomised trials. BMJ.

[5] Guyatt GH, Oxman AD, Kunz R, Falck-Ytter Y, Vist GE, Liberati A, Schünemann HJ (2008). Going from evidence to recommendations. BMJ.

[6] Velmahos GC, Toutouzas KG, Sillin LF, Chan L, Clark RE, Theodorou D, Maupin F (2004). Cognitive task analysis for teaching technical skills in an inanimate surgical skills laboratory. Am J Surg.

[7] Britt RC, Novosel TJ, Britt LD, Sullivan M (2009). The impact of central line simulation before the ICU experience. Am J Surg.

[8] Evans LV, Dodge KL, Shah TD (2010). Simulation training in central venous catheter insertion: improved performance in clinical practice. Acad Med.

[9] Smith CC, Huang GC, Newman LR (2010). Simulation training and its effect on long-term resident performance in central venous catheterization. Simul Healthc.

[10] Andreatta P, Chen Y, Marsh M, Cho K (2011). Simulation-based training improves applied clinical placement of ultrasound-guided PICCs. Support Care Cancer.

[11] Peltan ID, Shiga T, Gordon JA, Currier PF (2015). Simulation improves procedural protocol adherence during central venous catheter placement: a randomized controlled trial. Simul Healthc.

[12] Oh EJ, Lee JH, Kwon EJ, Min JJ (2020). Simulation-based training using a vessel phantom effectively improved first attempt success and dynamic needle-tip positioning ability for ultrasound-guided radial artery cannulation in real patients: an assessor-blinded randomized controlled study. PLoS One.

[13] Ma IW, Brindle ME, Ronksley PE, Lorenzetti DL, Sauve RS, Ghali WA (2011). Use of simulation-based education to improve outcomes of central venous catheterization: a systematic review and meta-analysis. Acad Med.

[14] Madenci AL, Solis CV, de Moya MA (2014). Central venous access by trainees: a systematic review and meta-analysis of the use of simulation to improve success rate on patients. Simul Healthc.

[15] Barsuk JH, McGaghie WC, Cohen ER, Balachandran JS, Wayne DB (2009). Use of simulation-based mastery learning to improve the quality of central venous catheter placement in a medical intensive care unit. J Hosp Med.

[16] Barsuk JH, McGaghie WC, Cohen ER, O'Leary KJ, Wayne DB (2009). Simulation-based mastery learning reduces complications during central venous catheter insertion in a medical intensive care unit. Crit Care Med.

[17] Stewart LA, Clarke M, Rovers M, Riley RD, Simmonds M, Stewart G, Tierney JF (2015). Preferred Reporting Items for Systematic Review and Meta-Analyses of individual participant data: the PRISMA-IPD Statement. JAMA.

[18] Mansfield PF, Hohn DC, Fornage BD, Gregurich MA, Ota DM (1994). Complications and failures of subclavian-vein catheterization. N Engl J Med.

[19] Blaivas M, Adhikari S (2009). An unseen danger: frequency of posterior vessel wall penetration by needles during attempts to place internal jugular vein central catheters using ultrasound guidance. Crit Care Med.

